# Diethyl Malonate-Based Turn-On Chemical Probe for Detecting Hydrazine and Its Bio-Imaging and Environmental Applications With Large Stokes Shift

**DOI:** 10.3389/fchem.2020.602125

**Published:** 2021-03-18

**Authors:** Jianbo Qu, Zhi-Hao Zhang, Haitao Zhang, Zhen-Tao Weng, Jian-Yong Wang

**Affiliations:** School of Light Industry and Engineering, Qi Lu University of Technology, Shandong Academy of Sciences, Jinan, China

**Keywords:** fluorescent probe, hydrazine, Stokes shift, gas detection, cell imaging

## Abstract

Diethyl malonate-based fluorescent probe **NE-N**_**2**_**H**_**4**_ was constructed for monitoring hydrazine (N_2_H_4_). The novel probe **NE-N**_**2**_**H**_**4**_ exhibits good properties, such as large Stokes shift (about 125 nm), good selectivity, and low cytotoxicity. This sensing probe **NE-N**_**2**_**H**_**4**_ can be operated to detect hydrazine in living HeLa cells. Especially after soaking in probe solution, the thin-layer chromatography (TLC) plate could detect the vapor of hydrazine. Therefore, the probe **NE-N**_**2**_**H**_**4**_ might be used to monitor hydrazine in biosamples and environmental problem.

## Introduction

Hydrazine (N_2_H_4_) and its substituted derivatives are usually applied in the aerospace industry as rocket propellant due to the distinctive properties of flammability and explosion (Serov and Kwak, 2010). This molecule N_2_H_4_ has also been employed as a catalyst, corrosion inhibitor, and reducing agent in many different fields including pharmaceutical, agricultural, and applied chemical industries (Kean et al., 2006; Khaled, 2006; Rosca and Koper, 2008). Due to its high toxicity, it is also considered as a terrible pollutant to creatures and humans, which could make the lungs, livers, and kidneys cancerous (Garrod et al., 2005). Hence, 10 ppb is the upper line (CDC, 1988). That is why it is important to develop good methods for sensing N_2_H_4_ in real-time detection and environmental pollution.

In modern times, chromatography–mass spectrometry, titrimetric, and electrochemical methods have been reported for monitoring N_2_H_4_ (Karimi-Maleh et al., 2014; McAdam et al., 2015). During the past few years, molecular probes have been developed for biological imaging with good properties of high sensitivity, large Stokes shift, good selectivity, good biocompatibility, and real-time detection, etc., which were regarded as the most practical method (Lakowicz, 2006; Li et al., 2014; Tang et al., 2015; Zhou X. et al., 2015).

During the last few decades, a series of turn-on probes were applied to detect N_2_H_4_ in living biosamples (Cui et al., 2014; Goswami et al., 2014a,b, 2015; Liu et al., 2014, 2019; Qian et al., 2014; Qu et al., 2014; Raju et al., 2014; Sun et al., 2014, 2015; Xiao et al., 2014; Jin et al., 2015; Nandi et al., 2015; Yu et al., 2015; Zhang et al., 2015; Zhou J. et al., 2015; Dai et al., 2016; Reja et al., 2016; Chen et al., 2017; Li et al., 2017, 2018, 2019; Ma et al., 2017; Mahapatra et al., 2017; Jung et al., 2019; Paul et al., 2019; Shi et al., 2019; Xing et al., 2019; Fang et al., 2020; Han et al., 2020; Hou et al., 2020; Vijay and Velmathi, 2020), a few of which were constructed by cleavage of C = C bond (Sun et al., 2014; Reja et al., 2016; Li et al., 2017, 2018, 2019; Liu et al., 2019; Hou et al., 2020). Many examples were developed by the deprotection group from the fluorescent group (Cui et al., 2014; Goswami et al., 2014a, 2015; Liu et al., 2014; Qian et al., 2014; Qu et al., 2014; Raju et al., 2014; Jin et al., 2015; Sun et al., 2015; Yu et al., 2015; Zhang et al., 2015; Zhou J. et al., 2015; Chen et al., 2017; Ma et al., 2017; Mahapatra et al., 2017; Shi et al., 2019; Xing et al., 2019; Fang et al., 2020; Vijay and Velmathi, 2020). Additionally, the rest of the fluorescent molecules were used to monitor N_2_H_4_ using the property of special nucleophilicity of N_2_H_4_ (Goswami et al., 2014b; Xiao et al., 2014; Nandi et al., 2015; Dai et al., 2016; Jung et al., 2019; Paul et al., 2019; Han et al., 2020). That is why it is necessary to construct a powerful molecule for monitoring N_2_H_4_ by the way of cleavage of C = C bond.

**Scheme 1:**
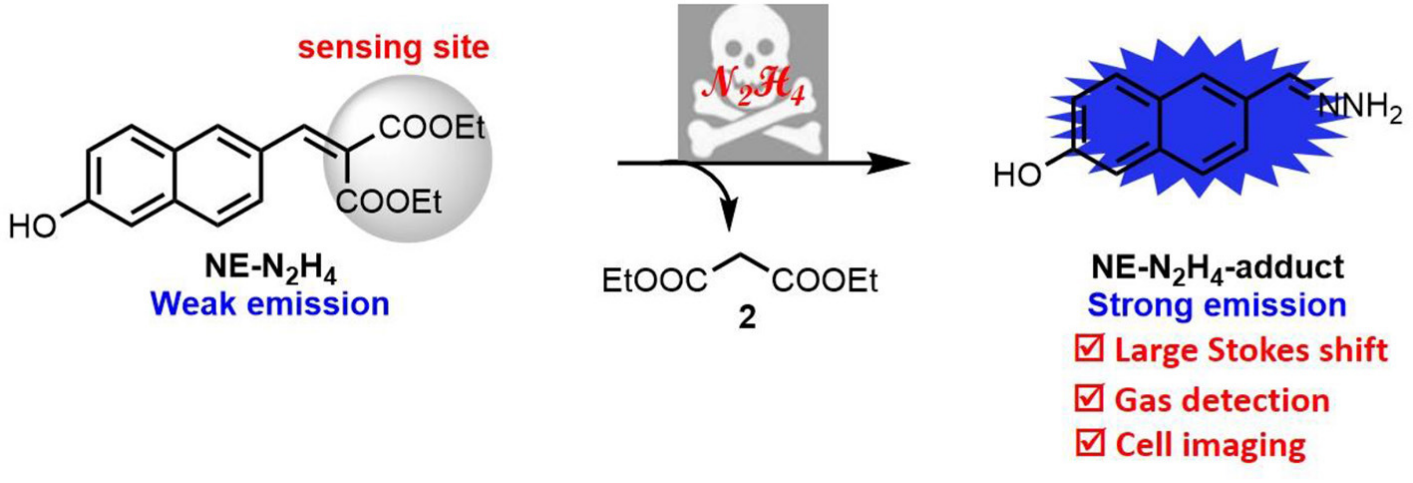
The molecular structure of **NE-N**_**2**_**H**_**4**_ and the proposed sensing mechanism.

In this report, a novel fluorescent probe, **NE-N**_**2**_**H**_**4**_, has been constructed to monitor N_2_H_4_ with improved properties including good selectivity, low cytotoxicity, and large Stokes shift over other analytes by cleavage of C = C bond (Scheme 1). The probe **NE-N**_**2**_**H**_**4**_ was applied to imaging N_2_H_4_ in living HeLa cells. Besides, the probe **NE-N**_**2**_**H**_**4**_ could monitor vapor of N_2_H_4_ by way of thin-layer chromatography (TLC) plate after soaking in solution of probe **NE-N**_**2**_**H**_**4**_. Therefore, this novel probe **NE-N**_**2**_**H**_**4**_ could be regarded as a powerful pool for monitoring N_2_H_4_ in biosystems and environmental pollution.

## Experiment

### Synthesis of Probe NE-N_2_H_4_

Here, 6-hydroxy-2-naphthaldehyde (1.0 mmol, 172.0 mg) and diethyl malonate (1.2 mmol, 192.2 mg) were added to EtOH (5.0 ml). Then, piperidine (1.2 mmol, 102.2 mg) was added to the above solution. After reacting at 25°C for 12 h, distilled H_2_O (10.0 ml) was added to the above reaction, which was extracted with dichloromethane (DCM) (50 ml) 3 times. All the extracts were washed with saturated aqueous sodium chloride solution and dried over MgSO_4_. The solid residue was dealt with flash column chromatography. The probe **NE-N**_**2**_**H**_**4**_ was obtained (83% yield). ^1^H NMR (400 MHz, CDCl_3_): 7.86 (s, 1H), 7.81 (s, 1H), 7.64 (d, *J* = 8.8 Hz, 1H), 7.52 (d, *J* = 8.8 Hz, 1H), 7.40 (dd, *J*_1_ = 2.0 Hz, *J*_2_ = 8.8 Hz, 1H), 7.09–7.04 (m, 2H), 4.41 (dd, *J*_1_ = 6.8, *J*_2_ = 14.0, 2H), 4.33 (dd, *J*_1_ = 7.2, *J*_2_ = 14.0, 2H), 1.37–1.32 (m, *6*H); ^13^C NMR (100 MHz, CDCl_3_): 167.5, 164.5, 155.3, 142.7, 135.6, 131.2, 130.7, 128.3, 128.0, 127.0, 125.9, 124.4, 118.8, 109.5, 61.9, 61.7, 14.2, 14.0; high-resolution mass spectrometry (HRMS) [electrospray ionization (ESI)] m/z calcd for C_18_H_18_O_5_ (M+H)^+^: 315.1230; found 315.1228.

### Vapor Gas Detection

TLC plates were soaked in the probe **NE-N**_**2**_**H**_**4**_ solution [0.1 mM, in dimethylsulfoxide (DMSO)]. After the **NE-N**_**2**_**H**_**4**_ probe-loaded TLC plates were dried over air-blast drying box, the plates were put onto a series of flasks with different concentrations of N_2_H_4_ for 10 min. Then, the color of probe-loaded TLC plates was observed under UV light of 365 nm.

### Cell Imaging

HeLa cells were grown in modified Eagle's medium (MEM) replenished with 10% fetal bovine serum (FBS) with the atmosphere of 5% CO_2_ and 95% air at 37°C for 24 h. The HeLa cells were washed with phosphate buffered saline (PBS) three times when used. HeLa cells were treated with **NE-N**_**2**_**H**_**4**_ (20.0 μM) for 30 min, then with N_2_H_4_ (200.0 μM) for 30 min at 37°C. The ideal fluorescence images were acquired with a Nikon A1MP confocal microscopy with the equipment of a CCD camera.

## Results and Discussion

### Design and Synthesis of Probe NE-N_2_H_4_

**Scheme 2:**
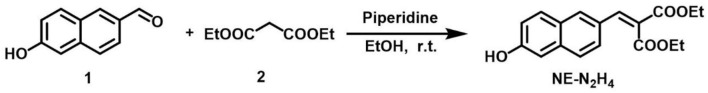
Synthesis of the fluorescent probe NE-N_2_H_4_.

As we all know, aldehyde group was easily reacted with nucleophile to construct C=C bond. Therefore, the simple compound of 6-hydroxy-2-naphthaldehyde was modified simply as the fluorescent group. The turn-on probe **NE-N**_**2**_**H**_**4**_ was developed by modifying a novel recognition site of diethyl malonate with functional aldehyde group in Scheme 2. The structure of the **NE-N**_**2**_**H**_**4**_ was characterized by ^1^H, ^13^C NMR, and HRMS (Supplementary Figures 8–10).

### The Spectral Property of Probe NE-N_2_H_4_

**Figure 1:**
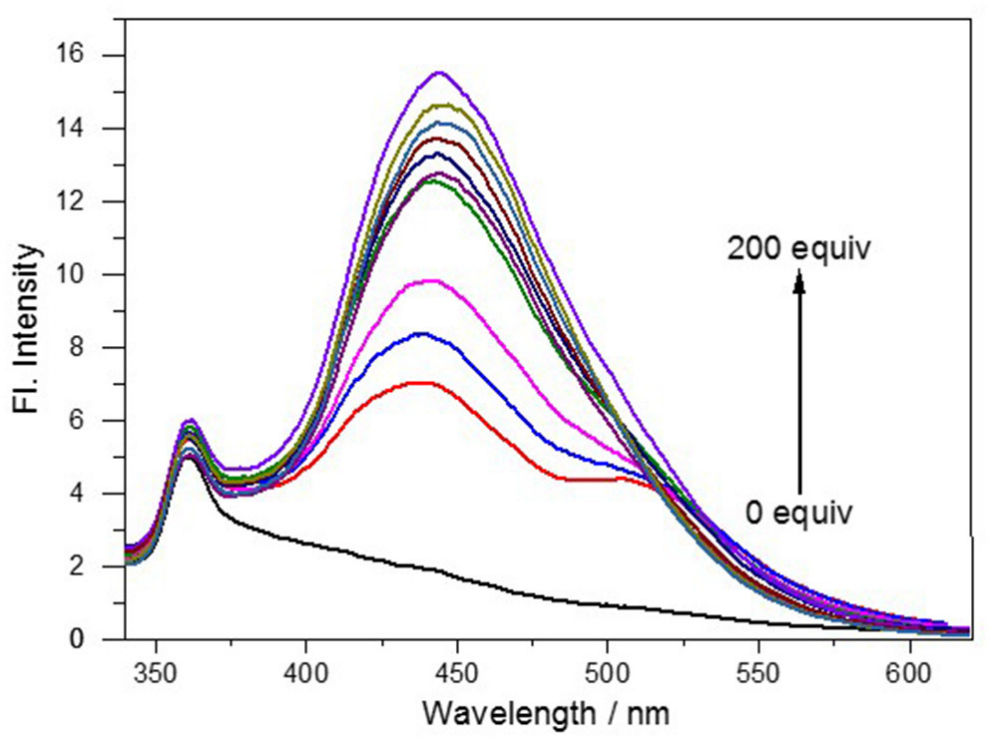
Fluorescence spectra of NE-N_2_H_4_ (10 μM) in pH 7.4 phosphate buffered saline (PBS)/dimethyl sulfoxide (DMSO0 (v/v = 1/1) in the absence or presence of N_2_H_4_.

This developed probe **NE-N**_**2**_**H**_**4**_ was applied to measure spectral properties with the addition of N_2_H_4_ including absorption spectroscopy and fluorescence spectroscopy. The probe **NE-N**_**2**_**H**_**4**_ exhibited no fluorescence under excitation at 320 nm without addition of N_2_H_4_ (Supplementary Figure 1, Figure 1). In contrary, strong fluorescence emission appeared at 445 nm after adding N_2_H_4_ to the solution of **NE-N**_**2**_**H**_**4**_, with a quantum yield of 0.35. When the addition of N_2_H_4_ was up to 200 equivalent, the fluorescence enhancement emerged to the high point (Figure 1). Therefore, the probe **NE-N**_**2**_**H**_**4**_ was easy to respond to N_2_H_4_, which was suitable for sensing N_2_H_4_ as a powerful pool with a large Stokes shift. The pH effect of PBS buffer was examined in Supplementary Figure 2. The fluorescent intensity increased from acid to basic rapidly. The main reason is that the nucleophilic substitution to the probe **NE-N**_**2**_**H**_**4**_ reacted easily in alkaline conditions.

### Mechanism

The sensing mechanism was examined by adding N_2_H_4_ to the solution of probe **NE-N**_**2**_**H**_**4**_. The reaction solution was detected by HRMS. When probe **NE-N**_**2**_**H**_**4**_ (20 μM) was treated with N_2_H_4_ (200 μM), a peak at m/z 187.0879 emerged in HRMS spectrum in accordance with the predicted **NE-N**_**2**_**H**_**4**_-**adduct** (Supplementary Figure 4). The **NE-N**_**2**_**H**_**4**_-**adduct** was constructed in one step easily characterized by ^1^H NMR and HRMS (Supplementary Figures 5, 6). Additionally, the absortion spectra of **NE-N**_**2**_**H**_**4**_ (10 μM) in absence or presence of N_2_H_4_ (10 equiv) and the synthetic **NE-N**_**2**_**H**_**4**_-**adduct** in pH 7.4 PBS/DMSO (v/v = 1/1) were listed in Supplementary Figure 3, which is consistent with the proposed mechanism (Scheme 1).

### Response Rate and Selectivity of Probe NE-N_2_H_4_

**Figure 2:**
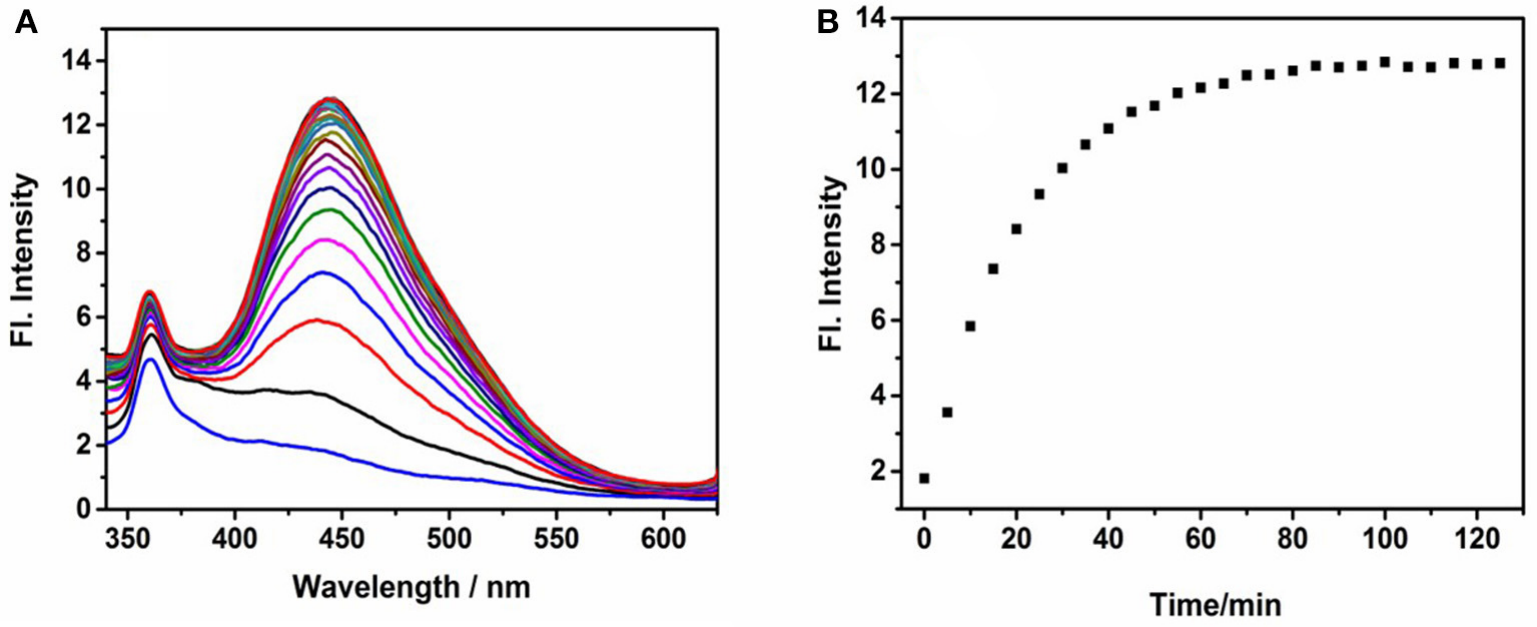
Fluorescence spectra of NE-N_2_H_4_ (10 μM) in pH 7.4 phosphate buffered saline (PBS)/dimethylsulfoxide (DMSO) (v/v = 1/1) in the absence or presence of N_2_H_4_ (10 equiv). **(A,B)** were depicted in Response Rate and Selectivity of Probe NE-N_2_H_4_.

**Figure 3:**
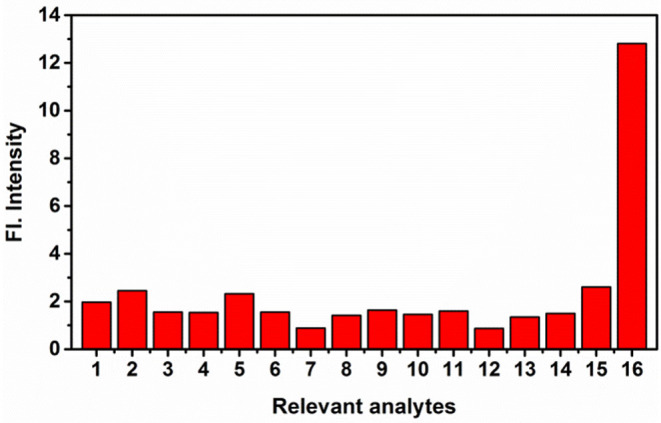
The fluorescence intensity of **NE-N**_**2**_**H**_**4**_ (10 μM) in the presence of various analytes (10 equiv) in phosphate buffered saline (PBS) buffer [pH 7.4 PBS/dimethylsulfoxide (DMSO) (v/v = 1/1)]. 1: None; 2: ${\text{SO}}_{3}^{2-}$; 3: ${\text{NO}}_{2}^{-}$; 4: ${\text{NO}}_{3}^{-}$; 5: I^−^; 6: Br^−^; 7: Fe^2+^; 8: H_2_O_2_; 9: NO; 10: Li^+^; 11: Zn^2+^; 12: Ni^2+^; 13: Cys; 14: GSH; 15: S^2−^; 16: N_2_H_4_.

The time course of probe **NE-N**_**2**_**H**_**4**_ was measured after the addition of N_2_H_4_ (10 equiv) (Figures 2A,B). Notably, the fluorescence enhancement was increased obviously as time goes on. That is to say, the sensing probe **NE-N**_**2**_**H**_**4**_ could be fit for imaging N_2_H_4_ in living cells. Another important factor is selectivity research of **NE-N**_**2**_**H**_**4**_ compared to other interfering species. It is very crucial whether the sensing molecule **NE-N**_**2**_**H**_**4**_ is suitable for cell imaging in the biosystem. The selectivity research was performed in Figure 3 over other competitive molecules. We find that fluorescence intensity showed almost no change after adding N_2_H_4_, when the solution of probe **NE-N**_**2**_**H**_**4**_ was added with other competitive molecules including ${\text{SO}}_{3}^{2-}$, ${\text{NO}}_{2}^{-}$, ${\text{NO}}_{3}^{-}$, I^−^, Br^−^, Fe^2+^, H_2_O_2_, NO, Li^+^, Zn^2+^, Ni^2+^, Cys, GSH, S^2−^, and N_2_H_4_. In conclusion, the sensing probe **NE-N**_**2**_**H**_**4**_ could be suitable for the response to N_2_H_4_ with good selectivity over other interfering molecules in the biosamples.

### Application in Gas Detection

**Figure 4:**
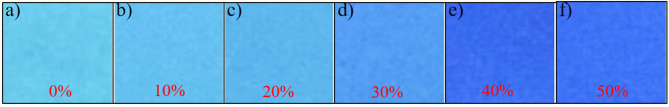
Photographs of thin-layer chromatography (TLC) plates, soaked in the solution of **NE-N**_**2**_**H**_**4**_, followed by addition of different amounts of hydrazine. **(a)** Water, **(b)** 10% N_2_H_4_, **(c)** 20% N_2_H_4_, **(d)** 30% N_2_H_4_, **(e)** 40% N_2_H_4_, **(f)** 50% N_2_H_4_.

According to the above research data, the application of gas detection was operated. The free TLC plates were soaked in the solution of **NE-N**_**2**_**H**_**4**_ (0.1 mM, in DMF). The TLC plates loaded with probe **NE-N**_**2**_**H**_**4**_ were prepared to discriminate N_2_H_4_ (gas) in different concentrations after drying with a vacuum dryer. Distinctive fluorescence color changes of plates were obtained under UV 365 nm light (Figure 4) after exposing TLC plates to the N_2_H_4_ (gas) for 10 min. Obviously, no obvious change was exhibited in the distilled water (Figure 4a). The experimental result indicated that the sensing probe **NE-N**_**2**_**H**_**4**_ may be a practical method to detect N_2_H_4_ in industrial pollution.

### Cytotoxicity and Imaging

Encouraged by the good fluorescent properties of probe **NE-N**_**2**_**H**_**4**_ including sensitive response, good selectivity, and large Stokes shift, a laser confocal microscope was applied to test the potential applications in cell imaging. The cytotoxicity of probe **NE-N**_**2**_**H**_**4**_was tested for imaging MTT assays in living cells. The living HeLa cells were operated for imaging fluorescent experiments by means of confocal laser scanning microscopy.

MTT assays were operated on living HeLa cells incubated with probe **NE-N**_**2**_**H**_**4**_ (see Supplementary Figure 4). The data indicated that this probe **NE-N**_**2**_**H**_**4**_ at different concentrations was almost nontoxic to the living cells [>90% HeLa cells survived after 24 h with **NE-N**_**2**_**H**_**4**_ (10.0 μM) incubation]. Therefore, this probe is fit for imaging N_2_H_4_ in living HeLa cells.

**Figure 5:**
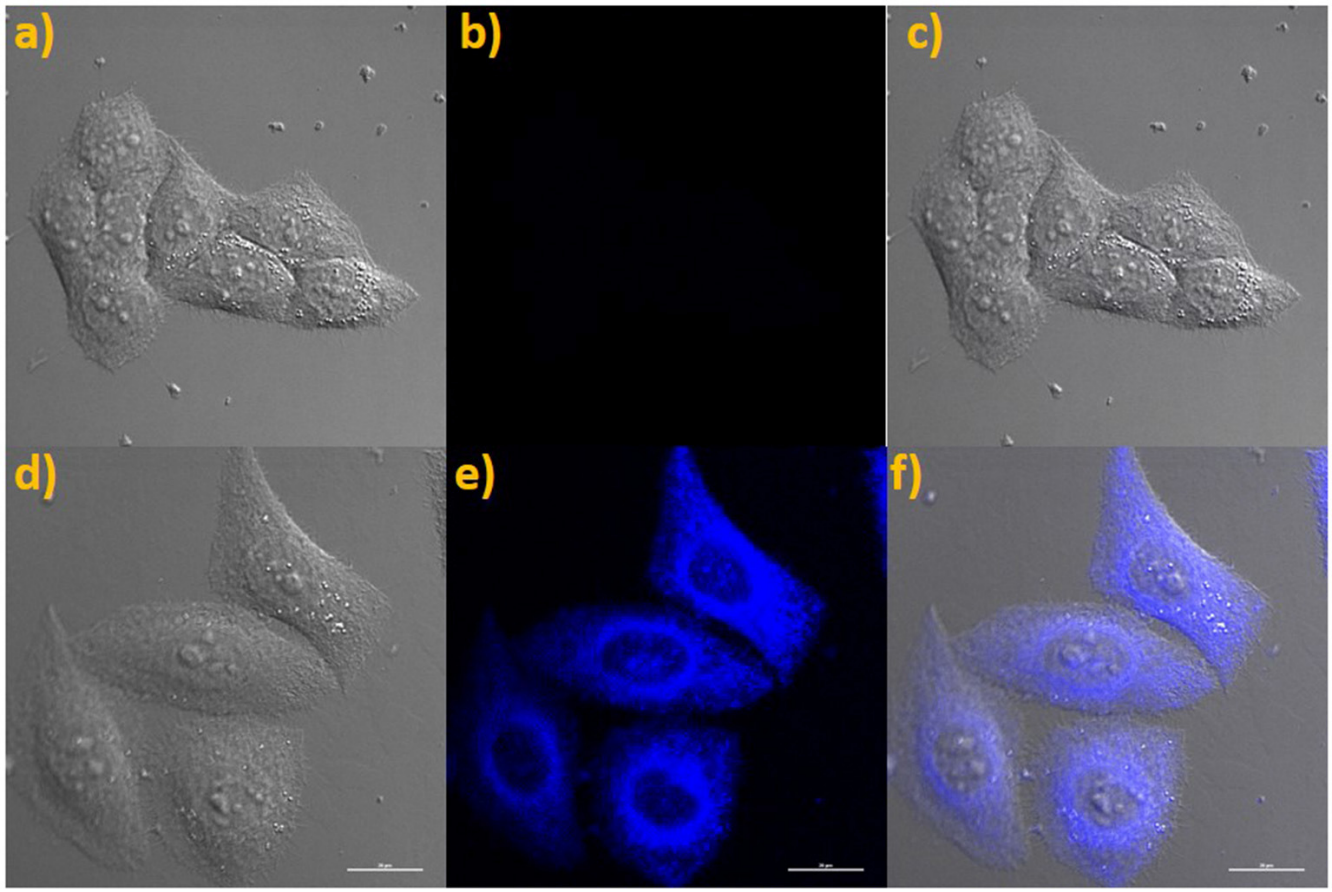
**(a)** Brightfield image of living HeLa cells costained only with **NE-N**_**2**_**H**_**4**_. **(b)** Fluorescence images of **(a)** from blue channel. **(c)** Overlay of **(a,b)**. **(d)** Brightfield image of living HeLa cells costained with **NE-N**_**2**_**H**_**4**_ and N_2_H_4_. **(e)** Fluorescence image of **(d)** from blue channel. **(f)** Overlay of the brightfield image **(d)** and blue channels **(e)**.

The probe **NE-N**_**2**_**H**_**4**_ was operated to incubate living HeLa cells for bioimaging of N_2_H_4_ due to the improved properties. Firstly, the solution of probe **NE-N**_**2**_**H**_**4**_ was used for incubating living HeLa cells for 30 min. No obvious fluorescence emerged in blue channel collected with Nikon A1MP confocal microscopy with a CCD camera (Figures 5a–c). Then, probe **NE-N**_**2**_**H**_**4**_ was used to incubate the living HeLa cells for 30 min and treated with N_2_H_4_ for another 30 min, obvious fluorescence exhibited in blue channel (Figures 5d–f). The experimental data indicated that the probe **NE-N**_**2**_**H**_**4**_ was fit for imaging N_2_H_4_ in living HeLa cells.

## Conclusion

In conclusion, an organic fluorescent probe has been constructed using diethyl malonate as a recognition site for sensing N_2_H_4_ with good selectivity and large Stokes shift (125 nm). This novel probe **NE-N**_**2**_**H**_**4**_ was developed for sensing N_2_H_4_ in living HeLa cells. In addition, this probe **NE-N**_**2**_**H**_**4**_ was applied for gas detection by probe-loaded TLC plate. The above results indicate that the probe **NE-N**_**2**_**H**_**4**_ may be powerful for monitoring N_2_H_4_ in biosystems and environmental problem.

## Data Availability Statement

The original contributions presented in the study are included in the article/Supplementary Materials, further inquiries can be directed to the corresponding author/s.

## Author Contributions

J-YW: synthesize and characterize the dyes. JQ: supervise the project, review, and edit manuscript. HZ: supervise the project, review, and edit manuscript. Z-HZ and Z-TW: design, synthesize, characterize the dyes, write and edit manuscript, and manage the research project. All authors: contributed to the article and approved the submitted version.

## Conflict of Interest

The authors declare that the research was conducted in the absence of any commercial or financial relationships that could be construed as a potential conflict of interest.
