# Supplementary material for: Diethyl Malonate-Based Turn-On Chemical Probe for Detecting Hydrazine and Its Bio-Imaging and Environmental Applications With Large Stokes Shift
Source: Front Chem. 2021 Mar 18;8:602125. doi: 10.3389/fchem.2020.602125 (PMC8012553; doi:10.3389/fchem.2020.602125)
Supplement: Supplementary file 1 [file Data_Sheet_1.docx]

*Supporting Information for*

Diethyl malonate based turn-on chemical sensor detecting hydrazine and its bio-imaging and environmental applications

Jianbo Qu, Zhi-Hao Zhang, Haitao Zhang, Zhen-Tao Weng, Jian-Yong Wang*

*School of Light Industry and Engineering, Qi Lu University of Technology (Shandong Academy of Sciences), Jinan, 250353, P. R., China,*

Email: [wjy@qlu.edu.cn](mailto:wjy@mail.ustc.edu.cn)

*Correspondence to: Jian-Yong Wang, School of Light Industry and Engineering, Qi Lu University of Technology(Shandong Academy of Sciences), Jinan, 250353, P. R., China, Email: wjy@qlu.edu.cn

**Table of contents**

Materials and instruments……..……………………………………………………S3

Cell culture….……..……………………………………………………….……….S3

Determination of the fluorescence quantum yield ……………………….……….S3

Cytotoxicity assays …………………………………………………… ….……….S4

Imaging N_2_H_4_ in living HeLa cells …………………………………………….. S4

Fig. S1…………………………… …………………………………………….….S6

Fig. S2….…………………………… …………………………………………….S6

Fig. S3….………………………………………………………………….……….S7

Fig. S4….………………………………………………………………….……….S7

Fig. S5….…………………………………………………………………………..S8

Fig. S6….…………………………………………………………………………..S8

Fig. S7….…………………………………………………………………………..S9

Fig. S8….…………………………………………………………………………..S9

Fig. S9….…………………………………………………………………………..S10

Fig. S10….………………………………………………………………………..S10

**Materials and instruments**

Unless otherwise noted, all reagents and materials were purchased from commercial company and used without further purification. Twice-distilled water was applied to all experiments. High-resolution electronspray (ESI-HRMS) mass spectra were examined from Bruker APEX IV-FTMS 7.0T mass spectrometer; NMR spectra were obtained from AVANCE III 400 MHz Digital NMR Spectrometer with TMS as an internal standard; Electronic absorption spectra were recorded on a LabTech UV Power spectrometer; Photoluminescent spectra were obtained with a HITACHI F4700 fluorescence spectrophotometer; The fluorescence images were collected with Nikon A1MP confocal microscopy with a CCD camera; The pH measurements were implemented on a Mettler-Toledo Delta 320 pH meter; analysis was exhibited on silica gel plates and column chromatography was carried out over silica gel (mesh 200-300). Both TLC and were purchased from the Qingdao Ocean Chemicals.

**Determination of the fluorescence quantum yield**

Fluorescence quantum yields were determined by using fluorescein (0.1 M in NaOH) according to previous report.^1^ The fluorescence quantum yield of compound **NE-N_2_H_4_** and **NE-N_2_H_4_ -adduct** was calculated according to the following equation :

$$\eta_{s}=\frac{A_{r}I_{s}n_{s}^{2}}{A_{s}I_{r}n_{r}^{2}}\eta_{r}(A\leq0.05)$$

In the equation, s and r represent the sample and the reference (fluorescein) molecule respectively, *η* represents the fluorescence quantum yield, *A* is the absorbance of molecules that were controlled below 0.05 at the excitation wavelength for both molecules in the experiment, *I* means the integrated emission area and *n* is the refractive index of the solvent.

Cell culture

The living HeLa cells were cultured in the Dulbecco’s modified Eagle’s medium (DMEM) supplemented with fetal bovine serum (10% FBS) under the atmosphere containing 5% CO_2_ and 95% air at 37 °C.

Cytotoxicity assays:

The living cells line were treated in DMEM (Dulbecco’s Modified Eagle Medium) supplied with fetal bovine serum (10%, FBS), penicillin (100 U/mL) and streptomycin (100 μg/mL) under the atmosphere of CO_2_ (5%) and air (95%) at 37 °C. The HeLa cells were then seeded into 96-well plates, and 0, 1, 5, 10, 20, 30 μM (final concentration) of the probe NE-N_2_H_4_ (99.9% DMEM and 0.1% DMSO) were added respectively. Subsequently, the cells were cultured at 37 °C in an atmosphere of CO_2_ (5%) and air (95%) for 24 hours. Then the HeLa cells were washed with PBS buffer, and DMEM medium (500 μL) was added. Next, MTT (50 μL, 5 mg/mL) was injected to every well and incubated for 4 h. Violet formazan was treated with sodium dodecyl sulfate solution (500 μL) in the H_2_O-DMF mixture. Absorbance of the solution was measured at 570 nm by the way of a microplate reader. The cell viability was determined by assuming 100% cell viability for cells without NE-N_2_H_4_.

**Imaging N_2_H_4_ in living HeLa cells**

HeLa cells were grown in modified Eagle’s medium (MEM) replenished with 10% FBS with the atmosphere of 5% CO_2_ and 95% air at 37 °C for 24 h. The HeLa cells were washed with PBS when used. HeLa cells treated with **NE-N_2_H_4_** (20.0 μM) for 30 min, then with N_2_H_4_ (200.0 μM) for 30 min at 37 °C. The ideal fluorescence images were acquired with a Nikon A1MP confocal microscopy with the equipment of a CCD camera.

**Reference**

1. J. N. Demas, A. Crosby, *J. Phys. Chem.*, 1971, **75**, 991-1024;


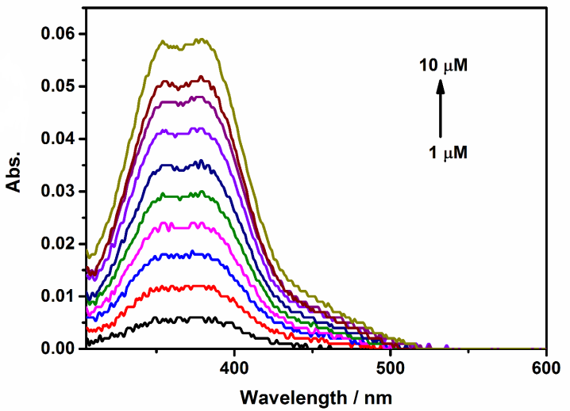


**Fig. S1** The absortion spectra of **NE-N_2_H_4_** (10 μM) in different concentration in pH 7.4 PBS/DMSO (v/v = 1/1).


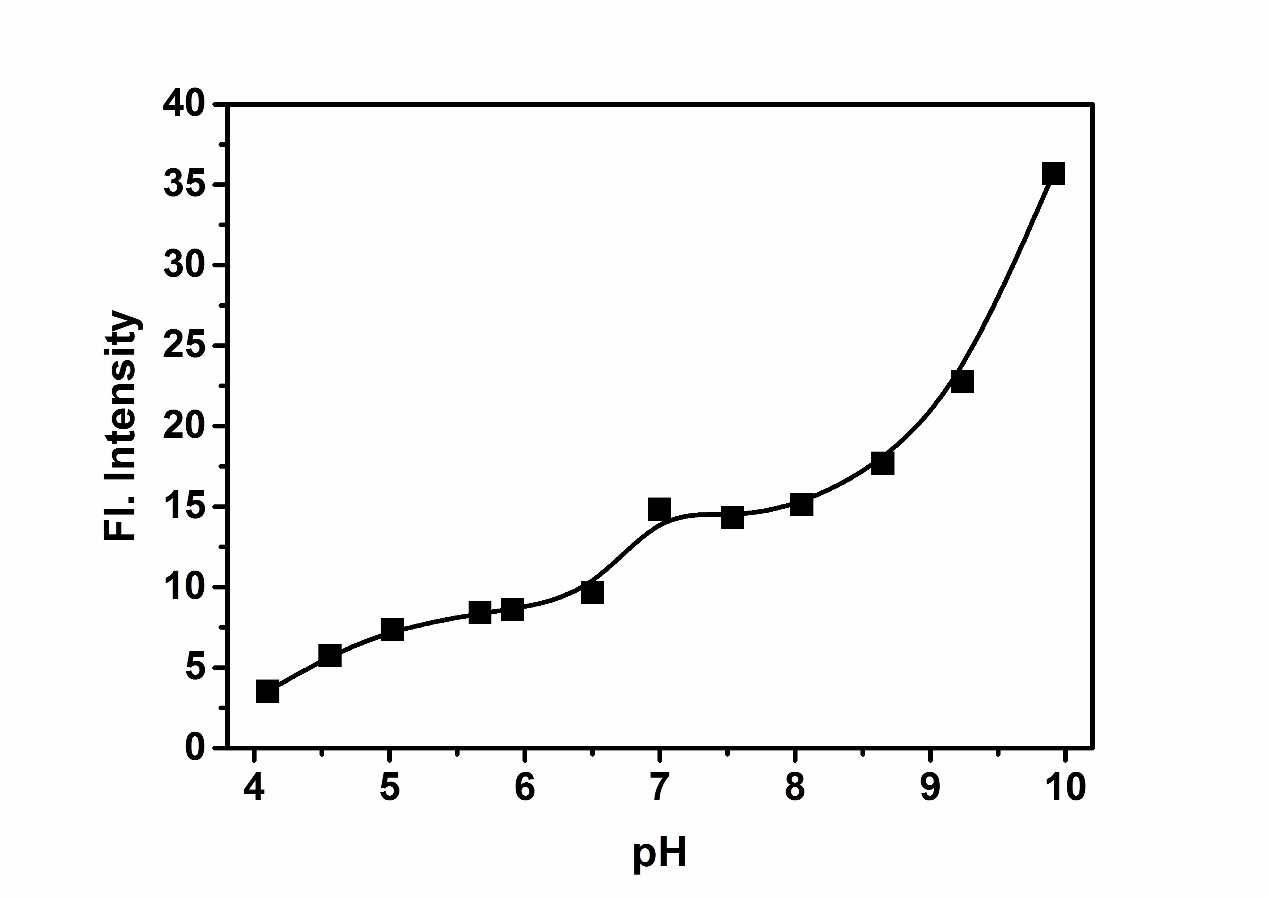


**Fig.** **S2** The pH-dependent fluorescence intensity of probe **NE-N_2_H_4_** (10 μM) in different pH PBS/DMSO (v/v = 1/1) with the addition of N_2_H_4_ (10 equiv).

**Fig. S3** The absortion spectra of **NE-N_2_H_4_** (10 μM) in absence or presence of N_2_H_4_ (10 equiv) and the synthetic **NE-N_2_H_4_**-**adduct** in pH 7.4 PBS/DMSO (v/v = 1/1).


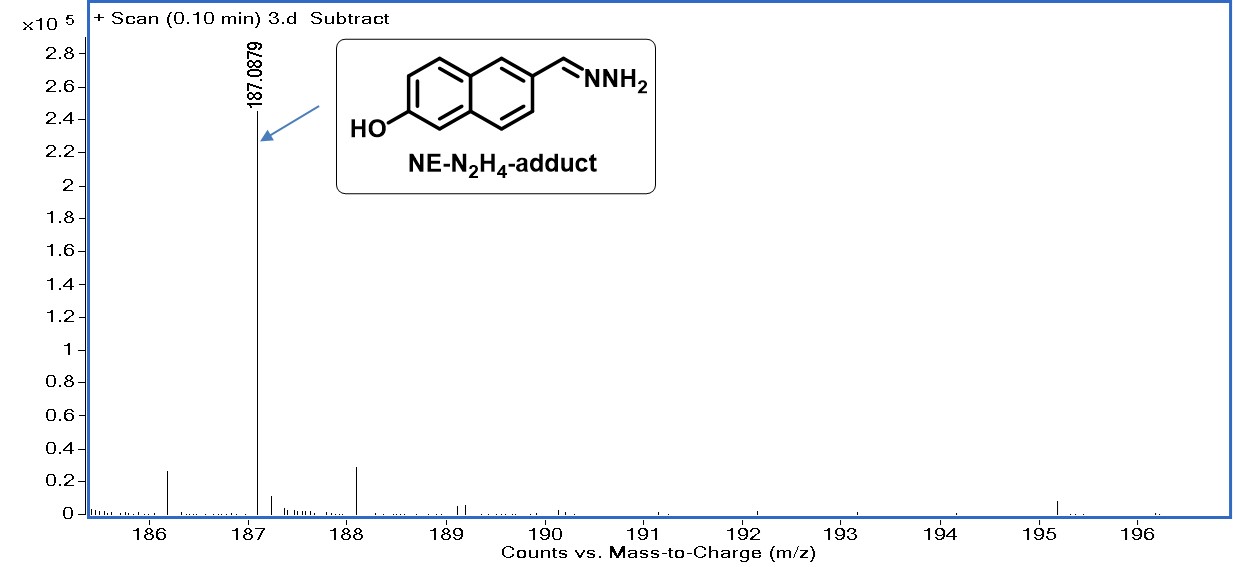


**Fig. S4**. HRMS (positive ion mode) spectrum of **NE-N_2_H_4_**. (20 μM) after treatment with N_2_H_4_ (200 μM) in pH 7.4 PBS/DMSO (1/1) for 20 min. The peak at m/z 187.0879 corresponds to **NE-N_2_H_4_**-**adduct**.

**
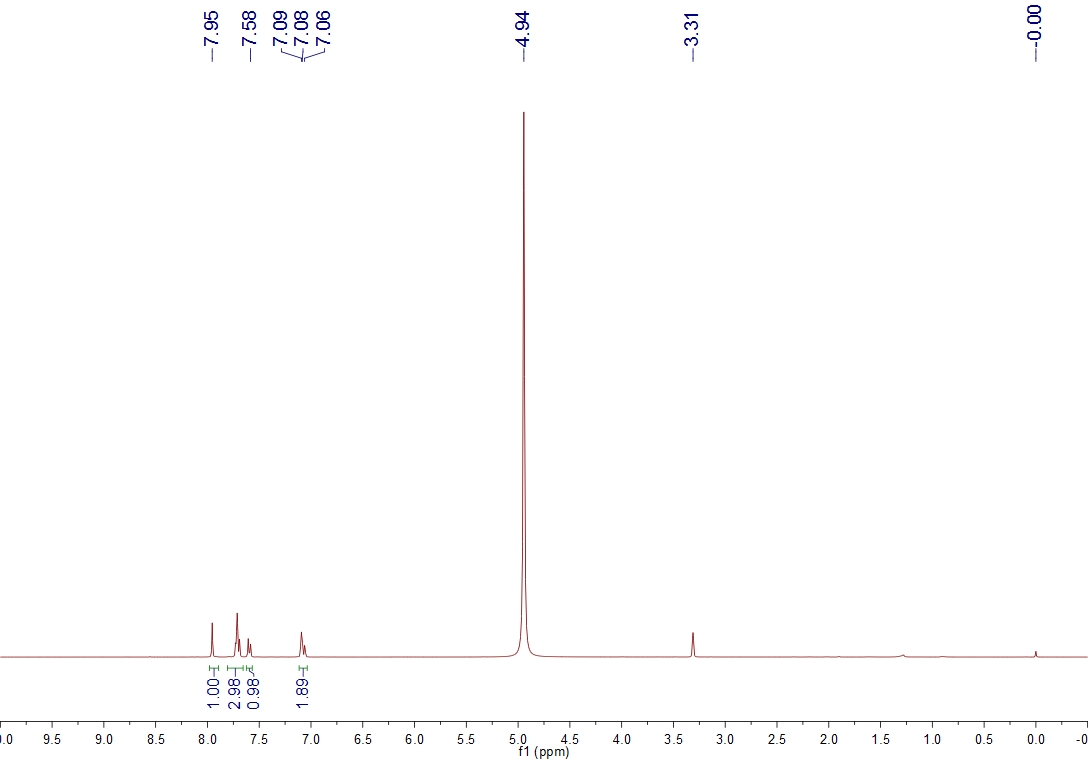
**

**Fig. S5**. ^1^H NMR (CD_3_OD) spectrum of the Synthetic **NE-N_2_H_4_**-**adduct**.

**Fig. S6**. HRMS (positive ion mode) spectrum of the Synthetic **NE-N_2_H_4_**-**adduct**. The peak at m/z 187.0864 corresponds to **NE-N_2_H_4_**-**adduct**.


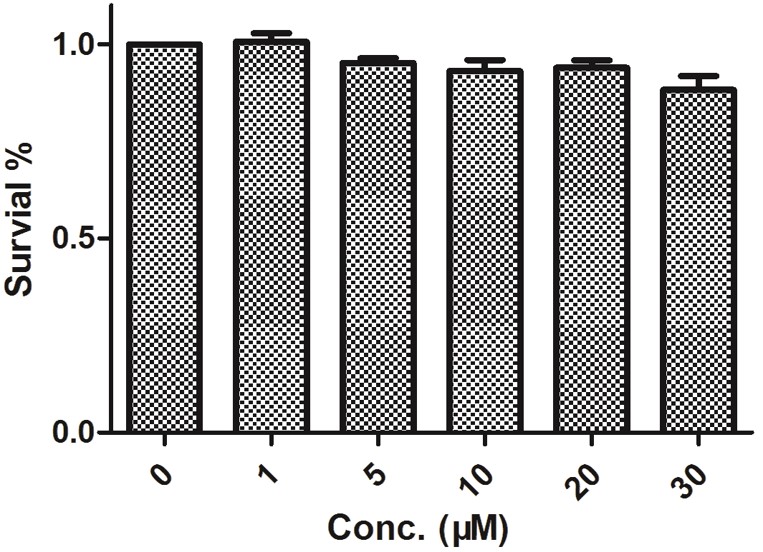


**Fig. S7** Cytotoxicity assays of **NE-N_2_H_4_** at different concentrations (0 μΜ; 1μΜ; 5 μΜ; 10 μΜ; 20 μΜ; 30 μΜ) for HeLa cells


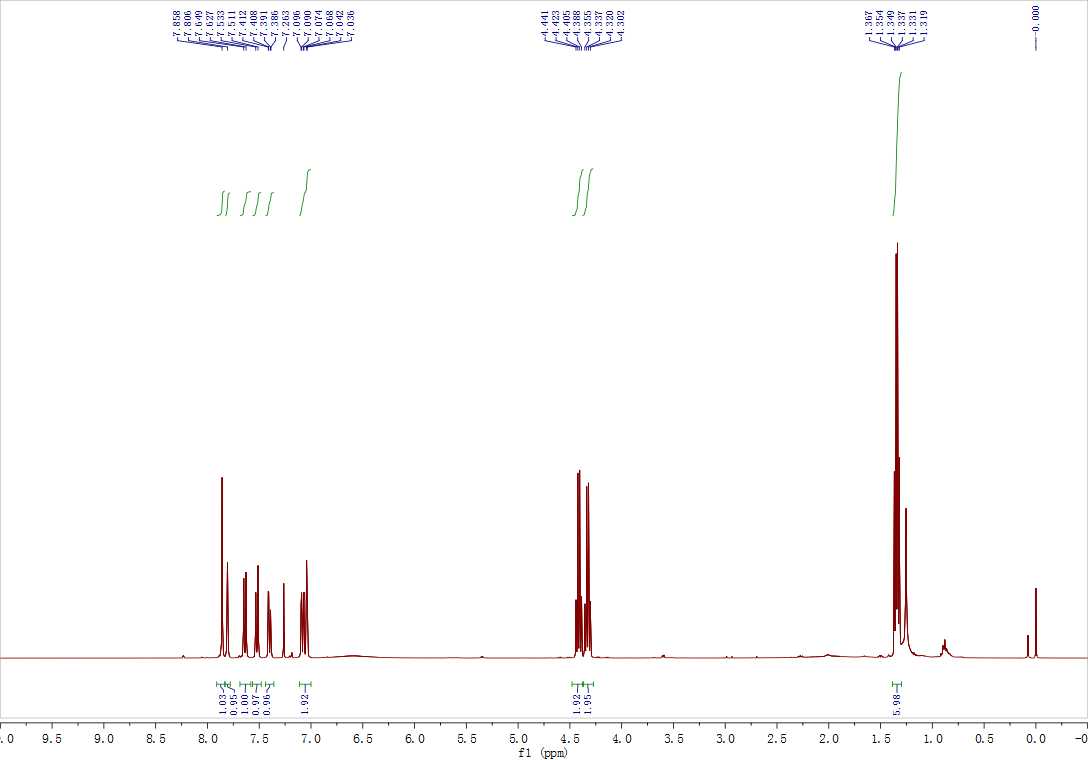


**Fig. S8**. ^1^H NMR (CDCl_3_) spectrum of **NE-N_2_H_4_.**


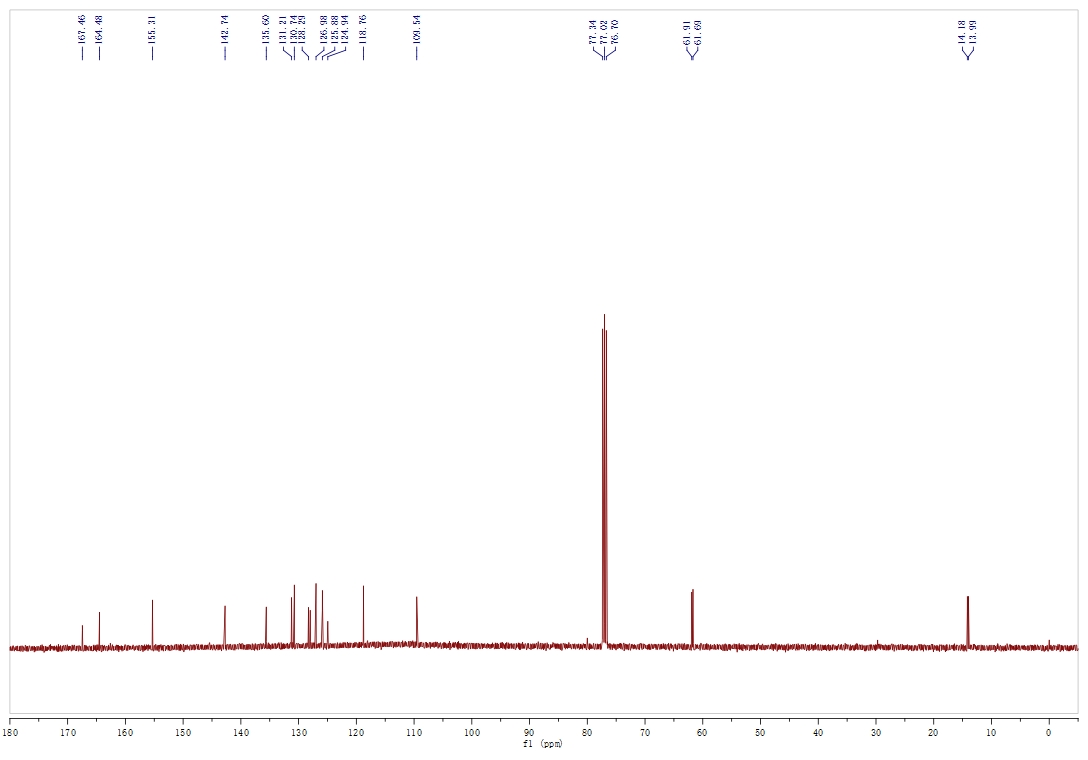


**Fig. S9**. ^13^C NMR (CDCl_3_) spectrum of **NE-N_2_H_4_.**


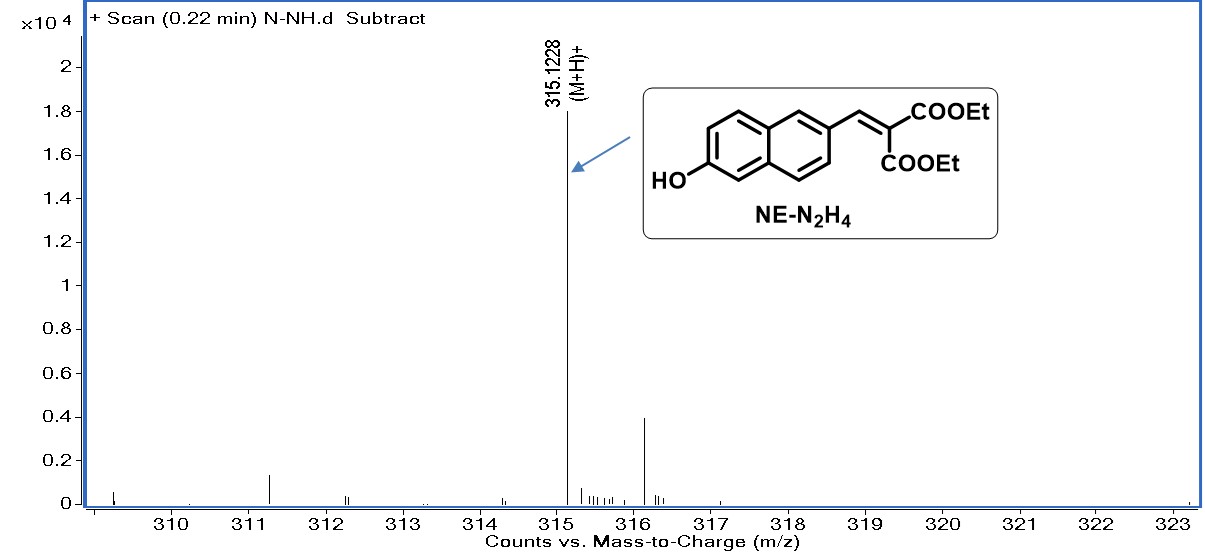


**Fig. S10**. HRMS (positive ion mode) spectrum of **NE-N_2_H_4_**.
