# Supplementary material for: Diethyl Malonate-Based Turn-On Chemical Probe for Detecting Hydrazine and Its Bio-Imaging and Environmental Applications With Large Stokes Shift
Source: Front Chem. 2021 Mar 18;8:602125. doi: 10.3389/fchem.2020.602125 (PMC8012553; doi:10.3389/fchem.2020.602125)

**Diethyl malonate based turn-on chemical probe for detecting hydrazine and its bio-imaging and environmental applications with large Stokes shift**

Jianbo Qu, Zhi-Hao Zhang, Haitao Zhang, Zhen-Tao Weng, Jian-Yong Wang*

Diethyl malonate based fluorescent probe **NE-N_2_H_4_** was constructed for monitoring hydrazine (N_2_H_4_). The novel probe **NE-N_2_H_4_** exhibited good properties, such as large Stokes shift (about 125 nm), good selectivity and low cytotoxicity. This turn-on probe **NE-N_2_H_4_** can be operated to detect hydrazine in the living HeLa cells. Especially, the TLC plate soaked in probe solution could detect the vapor of hydrazine. Therefore, the construted probe could be used to monitor hydrazine in biosamples and environmental problem.


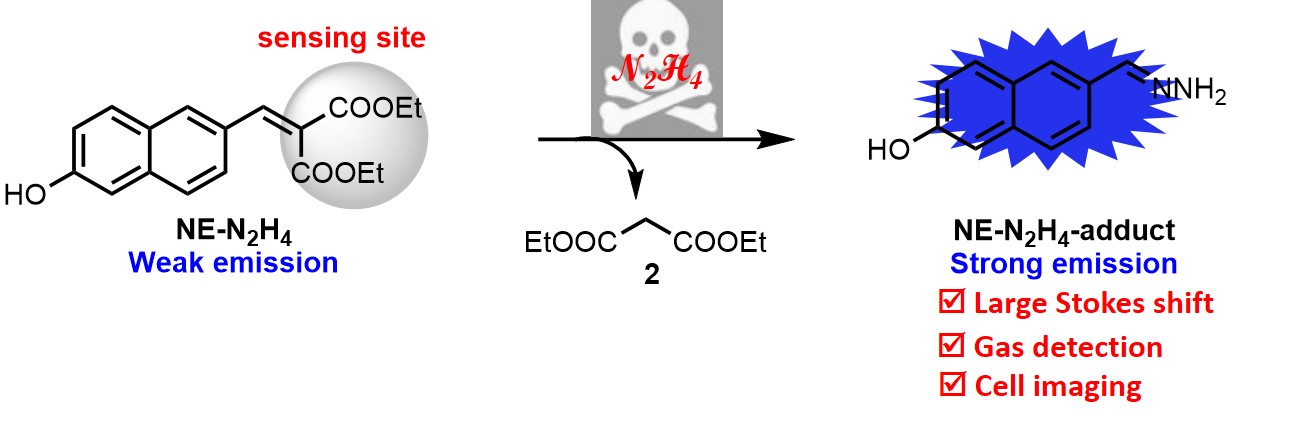

Supplement: Supplementary file 2 [file Data_Sheet_2.docx]
